# Hierarchical Fractal Interfaces in 3D-Printed Stiff–Soft Polymer Composites: Fracture Resistance and Toughening Mechanisms

**DOI:** 10.3390/polym18182230

**Published:** 2026-09-12

**Authors:** Pei-Rong Lin, Yung-Chu Tsai, Po-Yu Chen

**Affiliations:** Department of Materials Science and Engineering, National Tsing Hua University, Hsinchu 300044, Taiwan

**Keywords:** bio-inspired composites, fractal interface, multimaterial additive manufacturing, fracture resistance, crack deflection

## Abstract

Hierarchical hard–soft interfaces offer a route to fracture-resistant polymer composites without relying solely on intrinsically tough constituents. Here, Koch-curve-derived interfacial networks with fractal orders *n* = 0–3 were embedded as a compliant photopolymer phase within a rigid matrix and fabricated by multimaterial PolyJet printing. Pre-notched tensile tests, an eccentrically loaded single-edge-notch tension framework, digital image correlation, laser confocal microscopy, scanning electron microscopy, and crack-path box-counting analysis were used to determine how fractal order and pattern orientation govern fracture. At 0°, the *n* = 2 design increased peak nominal stress, nominal strain at fracture, tensile toughness, and crack-path length by approximately 2.2-, 1.7-, 3.6-, and 2.1-fold, respectively, relative to *n* = 0. Hierarchy also raised the conditional stress-intensity factor *K*_C_ from 9.78 to 13.3–13.6 MPa·m^1/2^ for *n* = 2–3. The representative *n* = 2 specimen exhibited a broader strain-redistribution zone than the representative *n* = 0 and *n* = 3 specimens, while the *n* = 2 architecture showed the most tortuous fracture morphology, whereas added hierarchy at *n* = 3 yielded diminishing benefits and coincided with reduced printing fidelity. Pattern orientation altered the balance between load transfer, deformation, and crack guiding. Toughening arose primarily from interfacial crack deflection and branching, assisted by crack-tip blunting, strain delocalization, and uncracked-ligament bridging. Overall, the second-order fractal architecture printed at 0° provides the best balance of load-bearing capacity, deformability, and fracture resistance, establishing fractal order and pattern orientation as practical design variables for tough multimaterial printed composites.

## 1. Introduction

The simultaneous achievement of high strength and high toughness remains a central challenge in structural-material design. Strategies that raise stiffness or strength often suppress deformation and increase sensitivity to flaws, whereas mechanisms that enhance energy dissipation can reduce load-bearing capacity [1]. Biological structural materials demonstrate that this trade-off can be moderated through architecture. Nacre, bone, teeth, and siliceous sponge skeletons combine comparatively stiff and compliant constituents across multiple length scales, using their interfaces to redistribute stress, divert cracks, and activate deformation over volumes much larger than the crack-tip process zone [2,3,4]. Their performance therefore arises not only from constituent properties but also from the spatial organization and mechanical contrast of the phases. Related analyses of biological hierarchy, nacre, and sponge biosilica further show that multiscale organization and compliant interfaces can combine load bearing with distributed damage [5,6,7,8,9,10,11,12].

Sutured and interdigitated interfaces are especially relevant to synthetic fracture-resistant composites. Natural and bio-inspired sutures increase interfacial path length and can tune stiffness, strength, and energy dissipation by changing hierarchical complexity [13,14,15]. In a hard–soft composite, elastic mismatch perturbs the local crack-tip field: a compliant phase can blunt the crack and deform, whereas the adjacent rigid phase maintains load transfer. Repeated interfaces can further force the crack to deflect or branch, increasing the effective fracture path and activating extrinsic shielding behind the advancing tip [3]. These mechanisms suggest that interfacial geometry may be treated as a design variable rather than as an unavoidable material boundary. Recent computational and experimental work on 3D-printed binary composites has further shown that the spatial placement of stiff and compliant phases relative to an edge crack can be deliberately optimized to tailor stiffness and toughness [16]. These principles have been resolved from tablet to structural scales and translated into synthetic nacre-like and weak-interface systems [17,18,19,20,21,22].

Fractal geometry provides a compact means of introducing controlled hierarchy. Previous fracture-oriented studies have considered a Koch-shaped metallic reinforcement embedded in a cementitious matrix [23], crack-path-engineered lattices [24], and programmable single-material metamaterials [25]. Fractal-inspired multimaterial polymer composites have also been realized by PolyJet printing: Wu et al. used Peano-curve-based rigid reinforcements within a flexible matrix and demonstrated that reinforcement hierarchy and alignment influence the tensile and compressive response [26]. Beyond these examples, engineered fractal or hierarchical structures have predominantly been developed for lightweight stiffness, shape reconfiguration, auxetic deformation, elastic-wave control, or compressive energy absorption [27,28,29,30,31], with structural hierarchy and fractal-like honeycombs also serving as routes to material efficiency and stiffness control [32,33]. However, the role of a compliant fractal network embedded within a rigid polymer matrix in controlling notch-driven crack propagation remains unclear. In particular, the coupled influence of fractal order and pattern orientation on crack deflection, strain redistribution, tensile fracture resistance, and the associated toughening mechanisms has not been systematically established.

Multimaterial additive manufacturing makes such architectures experimentally accessible. PolyJet material jetting can integrate rigid and compliant photopolymers within a single printed part and has been used to fabricate bio-inspired hierarchical multimaterial structures for tensile characterization, including full-field deformation measurements by digital image correlation [34]. Voxel-scale material placement in multimaterial jetting has recently been shown to widen the accessible range of strength, stiffness, and toughness in hard–soft photopolymer composites beyond manufacturer-preset material combinations [35]. Nevertheless, the same resolution that enables hierarchy also imposes a practical limit: as recursive features become finer, droplet spreading, voxel dimensions, and other process-related factors may alter line width, local phase fraction, and junction geometry. Dimensional accuracy in material jetting is known to depend on processing and geometric parameters, making fabrication fidelity an important consideration when increasingly fine architectures are compared [36,37]. In addition to dimensional accuracy, an interphase region forms between jetted dissimilar photopolymers and can measurably alter the mechanical response of multimaterial laminae, so that the effective local composition may deviate from the digital design [38]. Fabrication fidelity must therefore be evaluated together with mechanical performance, particularly when comparing successive fractal orders. The strong nonlinearity and formulation dependence of soft digital materials also make direct mechanical characterization essential in multimaterial printing [39]. Recent fracture experiments on jetted hard–soft interface composites further show that the interfacial toughness of such printed interfaces is rate-dependent, underscoring the need for direct fracture characterization of co-printed hard–soft systems [40].

The present study investigates Koch-curve-derived hard–soft interfaces fabricated by multimaterial PolyJet printing. In contrast to previous studies of architected interfaces, which have largely addressed suture, nacre-like, or single-scale patterned geometries, this work isolates recursive fractal order (*n* = 0–3) as the primary architectural variable, couples it systematically with pattern orientation (*θ* = 0°, 30°, 45°, and 60°), and quantifies fabrication fidelity at every hierarchical level, so that architectural and manufacturing effects can be separated when successive fractal orders are compared. To the authors’ knowledge, the coupling among recursive interfacial hierarchy, printing resolution, and tensile fracture behavior has not previously been established within a single systematic experimental framework for jetted hard–soft composites. Pre-notched tensile tests are combined with full-field digital image correlation (DIC) [41,42,43], crack-path box-counting analysis, confocal microscopy, and scanning electron microscopy (SEM). An ESE(T)-type geometry and the stress-intensity expression of ASTM E1922 are additionally adopted to obtain a conditional *K*_C_ for comparative ranking [44,45,46,47]. The objective is to determine how hierarchy and orientation govern the balance among load transfer, strain redistribution, crack-path tortuosity, and resistance to fracture, and to identify the mechanisms responsible for the optimum architecture.

## 2. Materials and Methods

### 2.1. Design of the Koch-Curve-Derived Composites

The architecture was motivated by the hierarchical sutures of ammonites, the square skeletal framework of glass sponges, and the cooperative hard–soft construction of nacre. A Koch-curve-derived ligament was used as a self-similar geometric unit within a square network. Parametric models were generated in Python 3.13.3 (Python Software Foundation, Wilmington, DE, USA), and the fractal order was varied from *n* = 0 to *n* = 3. The soft patterned phase was imported into Autodesk Netfabb Ultimate 2018 (Autodesk, Inc., San Rafael, CA, USA), and Boolean operations were used to generate the complementary rigid phase. The geometry evolved from a regular 5 × 5 grid at *n* = 0 to progressively finer hierarchical networks at *n* = 1–3. The pattern is derived from Koch recursion but is not a canonical Koch curve: line width at each order was solved from a fixed target soft-phase area rather than generated by a constant line-width scaling ratio.

**Figure 1:**
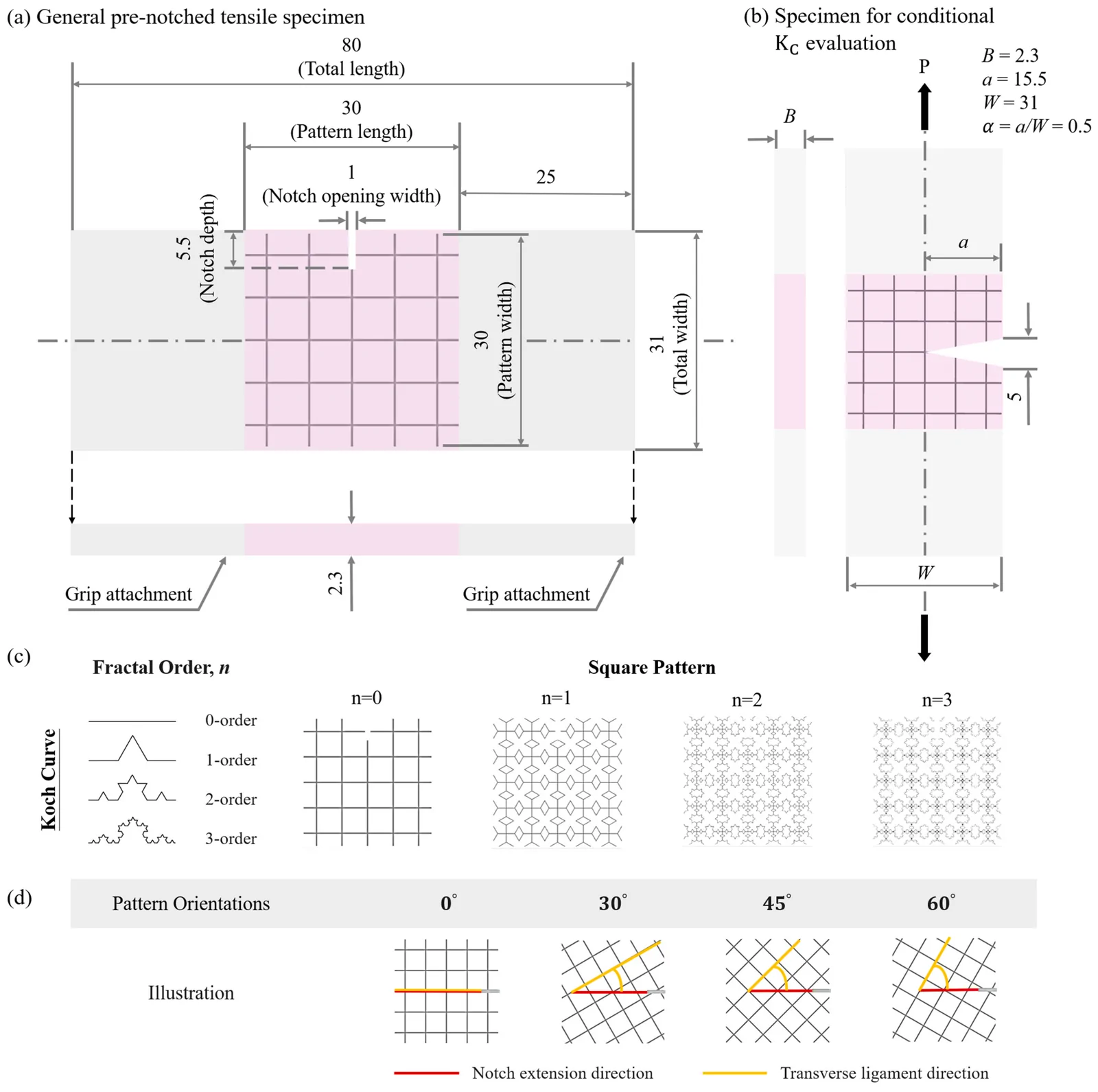
Specimen geometries and architectural variables. (**a**) General pre-notched tensile specimen with a notch depth of 5.5 mm and a notch opening width of 1 mm. (**b**) Modified ESE(T)-type specimen used for conditional *K_C_* evaluation, where B = 2.3 mm, a = 15.5 mm, W = 31 mm, *α* = *a*/*W* = 0.5, and the notch opening width is 5 mm. (**c**) Koch-curve-derived square networks with fractal orders *n* = 0–3. (**d**) Pattern orientation *θ*, defined as the angle between the notch-extension direction and the transverse-ligament direction. All dimensions are in mm.

Each specimen contained a 30 × 30 mm central patterned region embedded in an 80 × 31 mm tensile coupon with a thickness of 2.3 mm. The target soft-phase content was 10% of the patterned region. For the general pre-notched tensile tests, a rectangular edge notch with a depth of 5.5 mm and an opening width of 1 mm was introduced to localize crack initiation (Figure 1a). Pattern orientation *θ* was defined as the in-plane angle between the nominal notch-extension direction and the transverse ligament immediately ahead of the notch tip (Figure 1d). Four orientations—0°, 30°, 45°, and 60°—were produced by rotating the patterned region while holding the external dimensions, notch geometry, phase content, and material assignment constant. These angles sample the orientation space between ligaments aligned with the notch-extension direction (0°) and strongly inclined configurations at intervals fine enough to resolve the orientation dependence; 45° corresponds to the maximum-shear alignment of the ligament network relative to the tensile axis, and 30° and 60° bracket it symmetrically.

### 2.2. Materials and Multimaterial Additive Manufacturing

VeroPureWhite (Stratasys Ltd., Rehovot, Israel), a rigid opaque photopolymer, was assigned to the matrix. FLXA9895 (Stratasys Ltd., Rehovot, Israel), a rubber-like digital material with a nominal hardness of 90–96 Shore A, was assigned to the patterned phase. According to the manufacturer’s data sheets for the Stratasys J55 platform, VeroPureWhite exhibits a tensile strength of 40–55 MPa, an elongation at break of 5–20%, a modulus of elasticity of 2200–3000 MPa (ASTM D638 [48]), and a Shore hardness of 83–86 on the D scale, whereas FLXA9895, an Elastico-based digital material, exhibits a tensile strength of 10–13 MPa, an elongation at break of 75–105% (ASTM D412 [49]), a Shore hardness of 90–96 on the A scale, and a tensile tear resistance of 34–50 kg/cm [50,51]. Specimens were fabricated in a single build using a Stratasys J55 Prime PolyJet printer (Stratasys Ltd., Rehovot, Israel). In this material-jetting process, photopolymer droplets are deposited and ultraviolet-cured layer by layer, enabling rigid and compliant regions to be integrated without bonding separately manufactured parts. Droplets are cured immediately after deposition by the printer’s built-in ultraviolet lamps, whose exposure duration and intensity are machine-controlled for each material and are not user-adjustable. Specimens were printed in matte finish mode at the system’s high-quality setting, corresponding to a layer thickness of 18.75 μm and an in-plane resolution of 300 × 300 dpi.

After printing, residual support material was removed with a scraper. The specimens were then rinsed with water, wiped dry, and dried in an oven at 60 °C for 30 min. Mechanical testing was conducted at 25 °C approximately 24 h after printing.

The printed geometry was inspected before mechanical testing using a KEYENCE VK-X1000 series laser scanning confocal microscope (KEYENCE Corporation, Osaka, Japan). For each fractal order, three 0° specimens were examined and ten line-width measurements were taken per specimen, yielding 30 measurements per order. Measurements were taken normal to the local soft-phase line. Only in-plane width was quantified.

### 2.3. Mechanical Testing and Calculated Responses

Pre-notched tensile tests were performed with an Instron 3367 universal testing machine (Instron, Norwood, MA, USA) equipped with a 30 kN load cell. Specimens were clamped over 25 mm at each end, leaving the 30 mm patterned region between the grips. Load and crosshead displacement were recorded at 10 Hz. The fractal-order and orientation series were tested at a nominal strain rate of 1 × 10^−4^ s^−1^. Three specimens were tested per condition.

Nominal stress, *σ*, was calculated by dividing the applied load by the initial cross-sectional area, whereas nominal strain, *ε*, was calculated from the crosshead displacement divided by the initial gauge length. Peak nominal stress, *σ*_peak_, was defined as the maximum stress on the nominal stress–strain curve. Nominal strain at fracture, *ε_f_*, was defined as the nominal strain at final specimen failure. Tensile toughness was calculated as the area under the nominal stress–strain curve up to fracture. Crack-path length was measured from final fracture images. Tensile toughness was interpreted as a global energy-density measure, whereas crack-path length and crack-path box-counting dimension were used to characterize propagation-induced tortuosity; these quantities were therefore evaluated together with the conditional *K*_C_ rather than treated as interchangeable measures.

### 2.4. Conditional Stress-Intensity Factor

An eccentrically loaded single-edge-notch tension configuration was designed with reference to ASTM E1922 [44,45,46]. For the conditional *K_C_* evaluation, the general tensile-notch geometry was replaced by the modified geometry shown in Figure 1b. The specimen thickness *B* was 2.3 mm, the specimen width *W* was 31 mm, and the notch depth *a* was 15.5 mm, giving *α* = *a*/*W* = 0.5; the notch opening width was 5 mm. The nominal strain rate was 6.67 × 10^−4^ s^−1^ so that the time from zero load to peak load fell within the range specified by the adopted framework. The maximum applied stress-intensity factor was calculated from the peak load *P*_max_ as

(1) $$K_{C}=\frac{P_{max}}{B\sqrt{W}}\cdot \frac{\sqrt{\alpha }(1.4+\alpha )[3.97-10.88\alpha +26.25\alpha^{2}-38.90\alpha^{3}+30.15\alpha^{4}-9.27\alpha^{5}]}{{\left(1-\alpha \right)}^{3/2}}$$

where *B* is specimen thickness, *W* is specimen width, and *α* = *a*/*W*. The ESE(T) geometry and its stress-intensity expression were used as a consistent comparative framework. The material is fiber-free and ply-free, the notch has a finite root radius, and the thin specimens do not meet the plane-strain size requirements of ASTM E399 [52]. The resulting values are therefore reported as conditional *K*_C_ values and not as *K*_IC_ or translaminar toughness. In addition, the architected hard–soft composite is not homogeneous near the crack tip, so the measured value is geometry-dependent and is used only for comparative ranking within the common specimen geometry and procedure of this study. ASTM D5045 [53] provides the corresponding plane-strain framework for plastic materials, but its validity requirements are likewise not satisfied by the present thin, multimaterial specimens [53]. The materials, specimen geometries, and principal test conditions for both the pre-notched tensile and conditional *K_C_* tests are summarized in Table 1.

### 2.5. Crack-Path, Strain-Field, and Fracture-Surface Characterization

Final macroscopic crack paths were manually traced in ImageJ 1.54g (National Institutes of Health, Bethesda, MD, USA) to avoid confusing the printed soft network with an open crack. Connected primary branches were treated as one crack network, converted to binary images, and skeletonized to one-pixel-wide lines. Crack-path length was calculated from the skeletonized crack network. All crack paths were traced by the same operator using an identical workflow, and the path length is determined at the pixel resolution of the skeletonized images, so the comparisons among conditions are internally consistent. Box-counting analysis was performed with the FracLac plugin using four grid positions. The crack-path box-counting dimension, *D_B_*, was obtained from the slope of log N(r) against log(1/r), where N(r) is the number of boxes of size r intersecting the skeletonized crack [54,55,56,57]. Three specimens were analyzed per fractal order. *D_B_* was used only as a relative descriptor under a common image-processing workflow; values close to unity reflect the predominantly line-like nature of the macroscopic paths.

Two-dimensional DIC was conducted in MATLAB R2025a (The MathWorks, Inc., Natick, MA, USA) using Ncorr v1.2 [41,42,43]. A black speckle pattern was applied to the specimen surface. Images were sampled at 20 s intervals during early and intermediate deformation and at 10 frames s^−1^ near failure. The principal subset radius was 10 pixels, subset spacing was 1 pixel, and the y-direction strain component parallel to loading was evaluated. A strain-concentration onset was defined when a localized region reached at least 1.2 times the applied global strain and persisted for two consecutive frames. Owing to the extensive specimen matrix and the acquisition and processing demands of full-field DIC, one representative specimen was analyzed per condition; DIC was therefore used as qualitative and mechanistic evidence rather than for inferential statistics.

Fracture morphology was examined with the KEYENCE confocal microscope and a JEOL JSM-IT800 field-emission SEM (JEOL Ltd., Tokyo, Japan). Specimens were sputter-coated with platinum prior to observation, and SEM images were acquired at an accelerating voltage of 10 kV; overview fractographs were recorded at 100× magnification and detailed views at 300× magnification. Confocal observations captured macroscopic topology, whereas SEM was used to identify local deflection, surface steps, ligament stretching, and differences between rigid- and compliant-phase fracture regions.

### 2.6. Statistical Analysis

Mechanical results are reported as mean ± one standard deviation. One-way analysis of variance was applied within each comparison set, followed by Tukey’s honestly significant difference test when the omnibus test was significant. Pairwise comparisons were conducted among *n* = 0–3 and, within each fractal order, among *θ* = 0°, 30°, 45°, and 60°. Statistical significance was denoted by * *p* < 0.05, ** *p* < 0.01, and *** *p* < 0.001. Comparisons without significant differences were not marked. Analyses were performed in JMP Student Edition 19 (JMP Statistical Discovery LLC, Cary, NC, USA). For the fractal-order comparison at *θ* = 0°, the composite groups (*n* = 0–3) and the rigid (Hard) and compliant (Soft) single-material references were included in one comparison set. Three specimens were tested per condition, a sample size consistent with common practice in fracture studies of additively manufactured multimaterial polymers; accordingly, all comparative conclusions are based on the analysis of variance with post hoc testing described above rather than on individual comparisons.

## 3. Results and Discussion

### 3.1. Printing Fidelity and the Practical Hierarchy Limit

**Figure 2:**
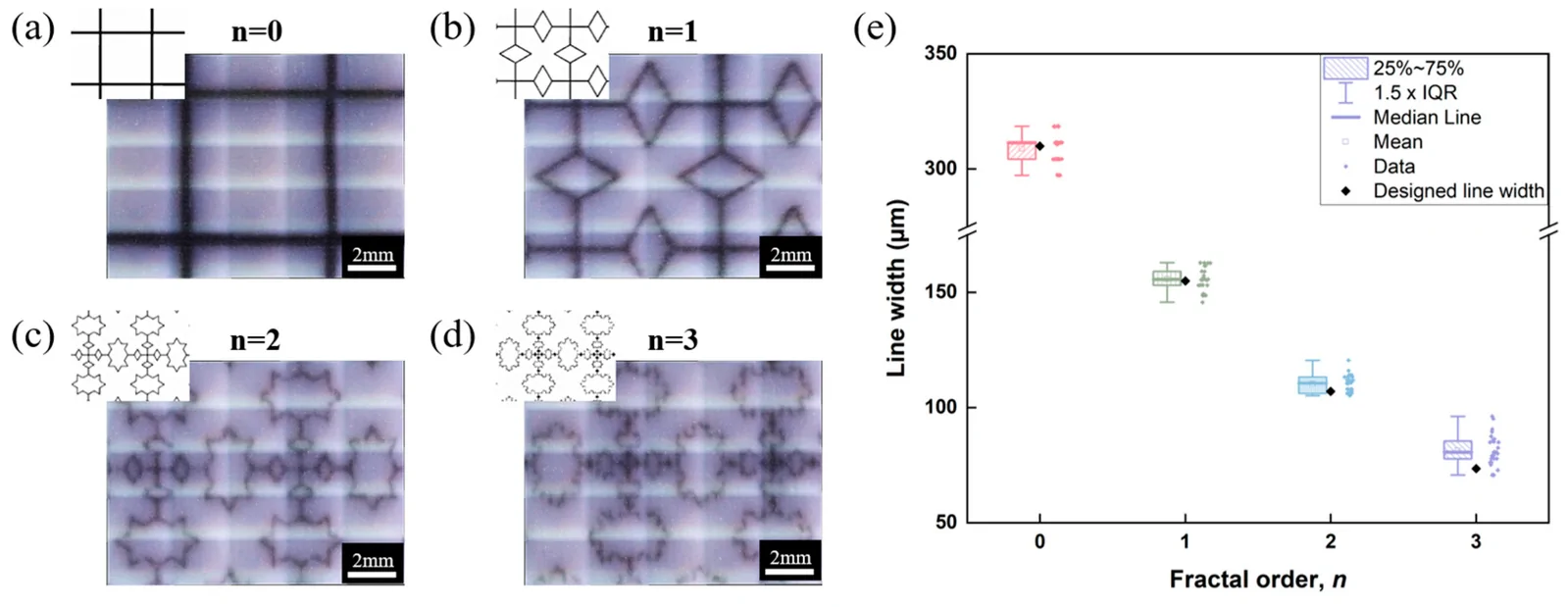
Printing fidelity of the Koch-curve-derived compliant networks. (**a**–**d**) Representative optical micrographs of the printed networks at fractal orders *n* = 0–3; the insets show the corresponding designed geometries, and all scale bars denote 2 mm. (**e**) Distributions of measured ligament widths and the corresponding designed values. Box boundaries denote the 25th and 75th percentiles, whiskers extend to 1.5 × the interquartile range, horizontal lines denote medians, open squares denote means, colored points denote individual measurements, and black diamonds denote designed widths. Each distribution contains 30 measurements obtained from three specimens with ten measurements per specimen.

The intended networks were formed at all four fractal orders, with no macroscopic unfilled ligaments or interfacial separation (Figure 2a–d). Feature definition nevertheless decreased as the target line width became smaller. The designed widths were 310, 155, 107, and 73.5 μm for *n* = 0–3, respectively. Corresponding measured values were 308.83 ± 6.45, 155.74 ± 4.83, 110.26 ± 3.63, and 81.41 ± 6.49 μm (Figure 2e and Table 2). The deviation remained below 1% at *n* = 0 and *n* = 1, rose to approximately 3% at *n* = 2, and reached approximately 11% at *n* = 3.

At the finer orders, the deviation was predominantly a broadening rather than a random undersizing of the compliant lines. A first-order estimate that treats area fraction as proportional to width at fixed centerline length gives actual soft-phase fractions of approximately 10.0%, 10.0%, 10.3%, and 11.1% for *n* = 0–3. This width-based value is an in-plane estimate at fixed centerline length and does not resolve through-thickness variation or sub-resolution mixing at the phase boundary. The approximately one-percentage-point offset at *n* = 3 is small, but the accompanying local merging and loss of edge definition show that *n* = 3 approaches the practical resolution limit of the present process [36]. Consequently, any apparent saturation from *n* = 2 to *n* = 3 should be interpreted as a coupled architectural and manufacturability effect, rather than as evidence that recursive hierarchy is intrinsically ineffective beyond the second order.

### 3.2. Fractal Order Redirects and Lengthens the Crack Path

**Figure 3:**
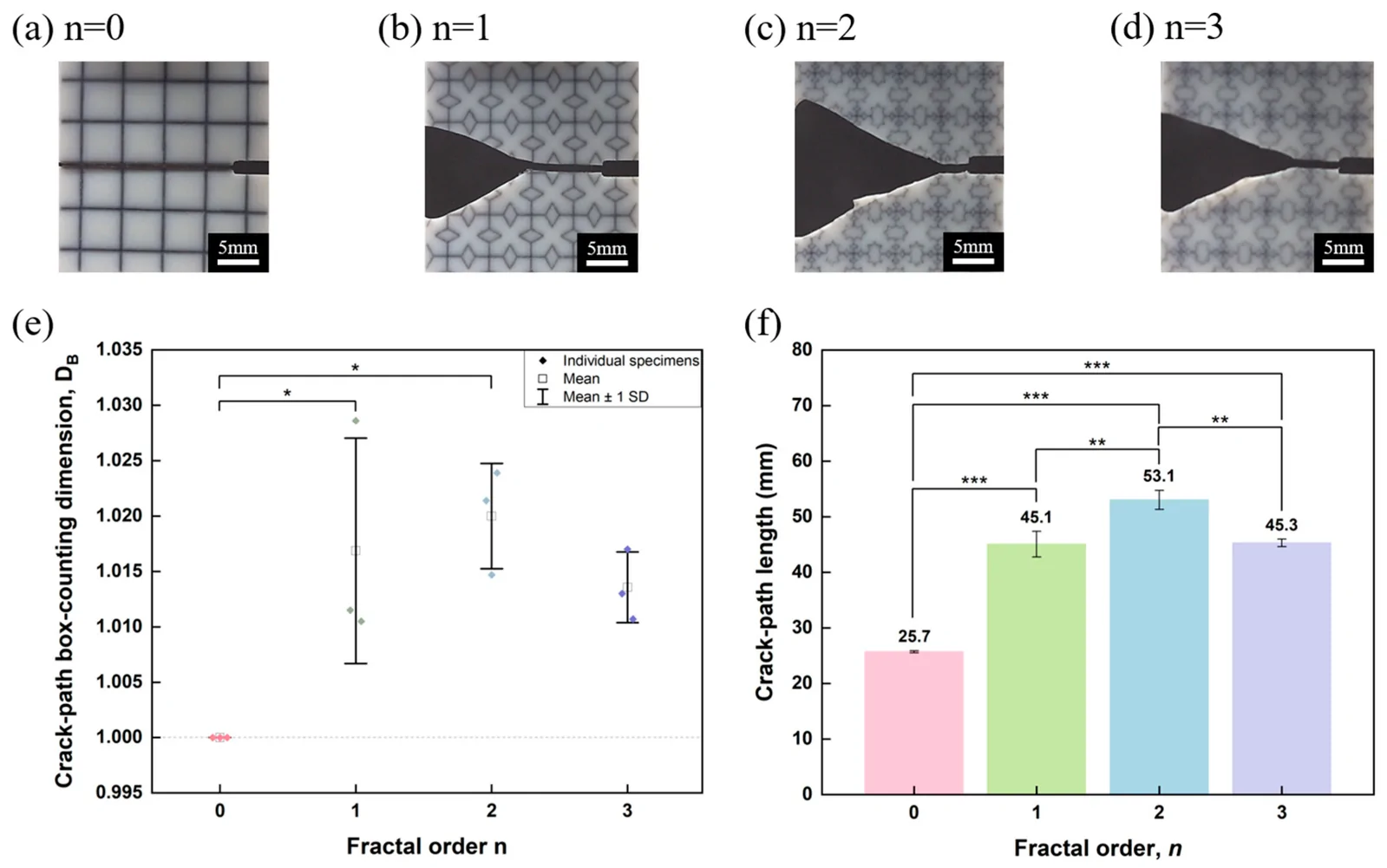
Fractal-order dependence of crack propagation. (**a**–**d**) Representative final crack paths for fractal orders *n* = 0–3. (**e**) Crack-path box-counting dimension, *D_B_*. Colored diamonds represent individual specimens, open squares represent the mean, and error bars indicate mean ± one standard deviation; the dashed horizontal line denotes *D_B_* = 1. (**f**) Crack-path length. Values in (**f**) are mean ± one standard deviation from three specimens per fractal order. Scale bars: 5 mm. Statistical significance is indicated by * *p* < 0.05, ** *p* < 0.01, and *** *p* < 0.001.

The *n* = 0 grid established a comparatively direct, architecture-guided fracture path. During propagation, a visible crack followed the central compliant line and reached final failure with little macroscopic branching. Introducing hierarchy altered this response. At *n* = 1, the final path separated into upper and lower branches; at *n* = 2, branching and tortuosity were most pronounced; and at *n* = 3, branching remained evident but was less extensive than at *n* = 2 (Figure 3a–d). Thus, increasing order did not produce a monotonic increase in macroscopic complexity.

This ranking was supported by two complementary geometric measures. Crack-path length increased from 25.7 mm at *n* = 0 to 45.1, 53.1, and 45.3 mm at *n* = 1, *n* = 2, and *n* = 3, respectively (Figure 3f). The crack-path box-counting dimension, *D_B_*, increased from the line-like reference value of 1.0000 at *n* = 0 to 1.0169, 1.0200, and 1.0136 for *n* = 1–3 (Figure 3e). Although the absolute increments in *D_B_* are small, the standardized extraction and skeletonization workflow provides a consistent relative descriptor. Across the four order means, crack-path length and tensile toughness followed the same rank order, and both crack-path length and *D_B_* reached their maxima at *n* = 2. These parallel trends support a mechanistic association between propagation-induced tortuosity and energy dissipation, but are not interpreted as a statistically established correlation because only four fractal-order means were compared. Fractal characteristics of fracture are also receiving renewed theoretical attention: crack networks in nacreous architectures have recently been shown to develop fractal patterns as an emergent failure mode arising from microstructural randomness [58].

### 3.3. Fractal Order Improves the Tensile Fracture Response

**Figure 4:**
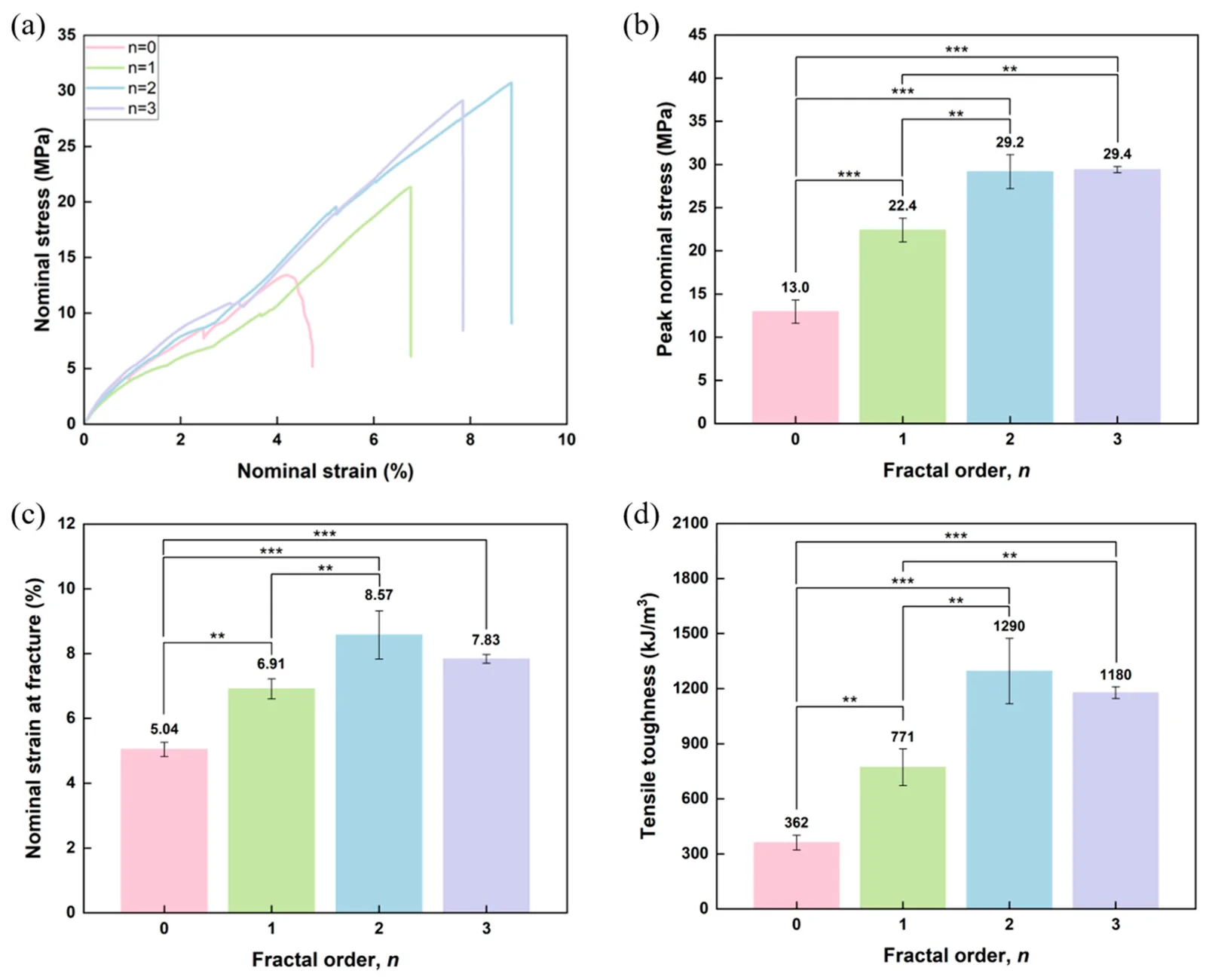
Effect of fractal order on the pre-notched tensile response at *θ* = 0°. (**a**) Representative nominal stress–strain curves for each fractal order. (**b**) Peak nominal stress, (**c**) nominal strain at fracture, and (**d**) tensile toughness. Bars show mean ± one standard deviation from three specimens per fractal order. Statistical significance is indicated by ** *p* < 0.01 and *** *p* < 0.001.

Representative nominal stress–strain curves for the four architectures are shown in Figure 4a. Hierarchy simultaneously increased the stress level and the deformation sustained before failure. Peak nominal stress rose from 13.0 MPa at *n* = 0 to 22.4 MPa at *n* = 1 and then reached 29.2 and 29.4 MPa at *n* = 2 and *n* = 3, respectively (Figure 4b). The nearly identical values at the two highest orders indicate a strength plateau. Nominal strain at fracture followed a different non-monotonic trend, increasing from 5.04% at *n* = 0 to 6.91% at *n* = 1 and reaching 8.57% at *n* = 2 before decreasing slightly to 7.83% at *n* = 3 (Figure 4c).

Because tensile toughness integrates both stress and strain, it most clearly exposed the optimum hierarchy. Toughness increased from 362 kJ m^−3^ at *n* = 0 to 771 kJ m^−3^ at *n* = 1, peaked at 1290 kJ m^−3^ at *n* = 2, and decreased modestly to 1180 kJ m^−3^ at *n* = 3 (Figure 4d). Relative to *n* = 0, *n* = 2 increased peak nominal stress, nominal strain at fracture, tensile toughness, and crack-path length by approximately 2.2-, 1.7-, 3.6-, and 2.1-fold, respectively. The *n* = 2 architecture therefore provided the best combined load-bearing, deformation, tensile-toughness and crack-path-extension response.

These responses show why no single metric should be used to define the optimum. Peak nominal stress alone would treat *n* = 2 and *n* = 3 as equivalent, whereas nominal strain at fracture, tensile toughness, crack-path length, and crack-path box-counting dimension consistently identify *n* = 2 as the strongest overall design. Conditional *K*_C_ is considered separately because it emphasizes peak-load notch resistance rather than the full propagation history.

### 3.4. Strain Redistribution and Fracture-Surface Evidence

**Figure 5:**
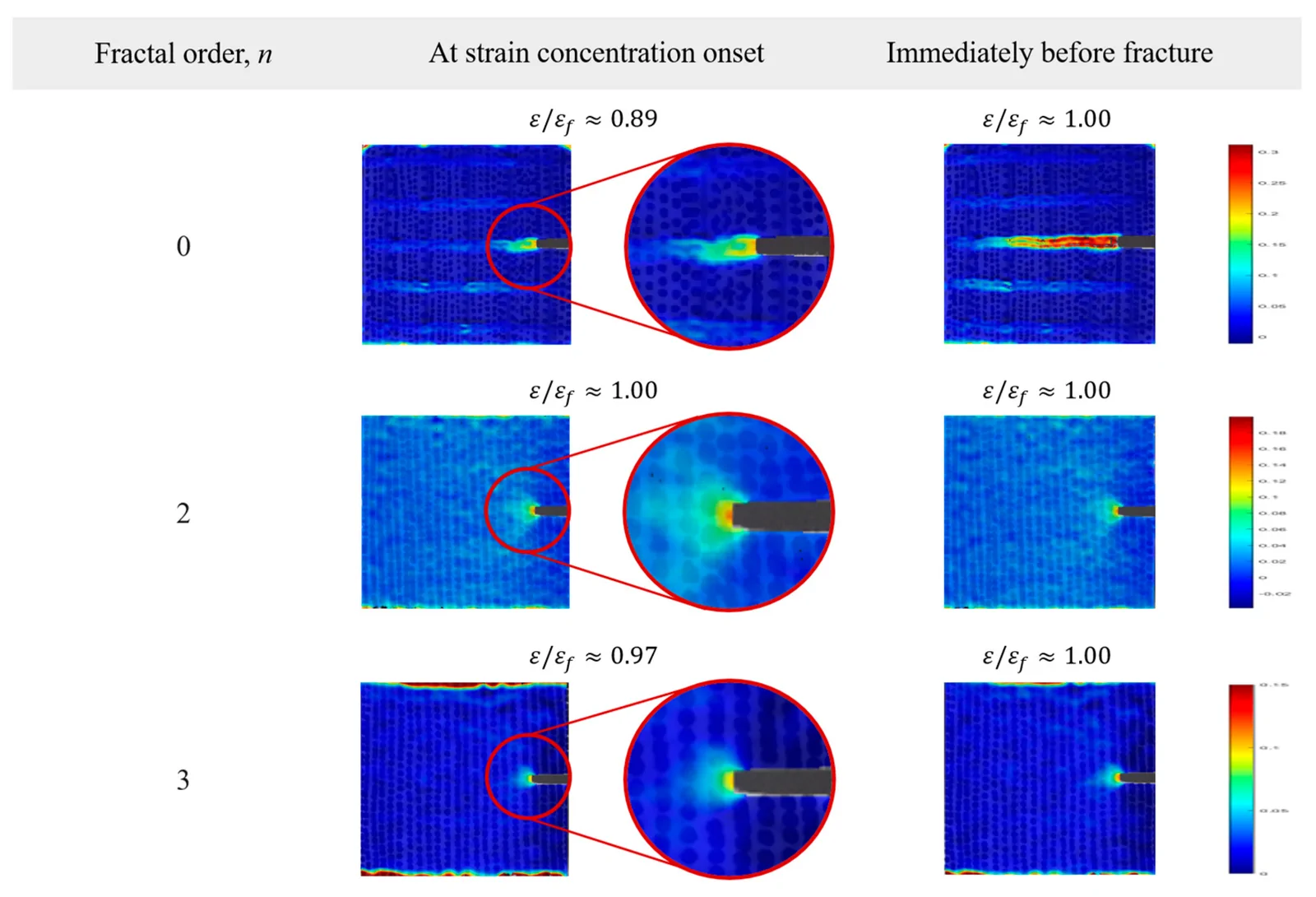
Strain-field evolution as a function of fractal order. Loading-direction strain maps, *ε_yy_*, are shown for representative specimens with *n* = 0, 2, and 3 at strain-concentration onset (**left**) and immediately before fracture (**right**). The normalized global strain *ε*/*ε_f_* is indicated above each map, where *ε_f_* is the nominal strain at fracture of the corresponding representative specimen. Onset was defined as the first localized region *near* the notch tip or expected crack path that reached *ε_yy_*_,*local*_ ≥ 1.2*ε_global_* for at least two consecutive frames. Circular insets enlarge the notch-tip field at onset. A common color scale is used for the two stages within each fractal order; scale limits differ among orders, so comparisons emphasize spatial distribution and temporal evolution rather than absolute color intensity. One representative specimen is shown per condition.

DIC revealed a transition from a narrow, architecture-guided localization band to a broader deformation zone (Figure 5). In *n* = 0, the defined strain-concentration onset occurred at ε/*ε_f_* ≈ 0.89, after which a narrow band intensified along the notch-extension direction. For *n* = 2, a spatially broader field persisted until ε/*ε_f_* ≈ 1.00, indicating that pronounced localization was delayed to the final stage of loading. The *n* = 3 specimen showed an intermediate response, with onset at ε/*ε_f_* ≈ 0.97 and a more localized notch-tip field than *n* = 2. These representative fields qualitatively connect the higher nominal strain at fracture of *n* = 2 with suppression of one dominant strain band. They are consistent with the flaw-tolerance and stress-delocalization effects reported for hierarchical architectures [59,60], but should not be interpreted as statistically replicated onset measurements.

**Figure 6:**
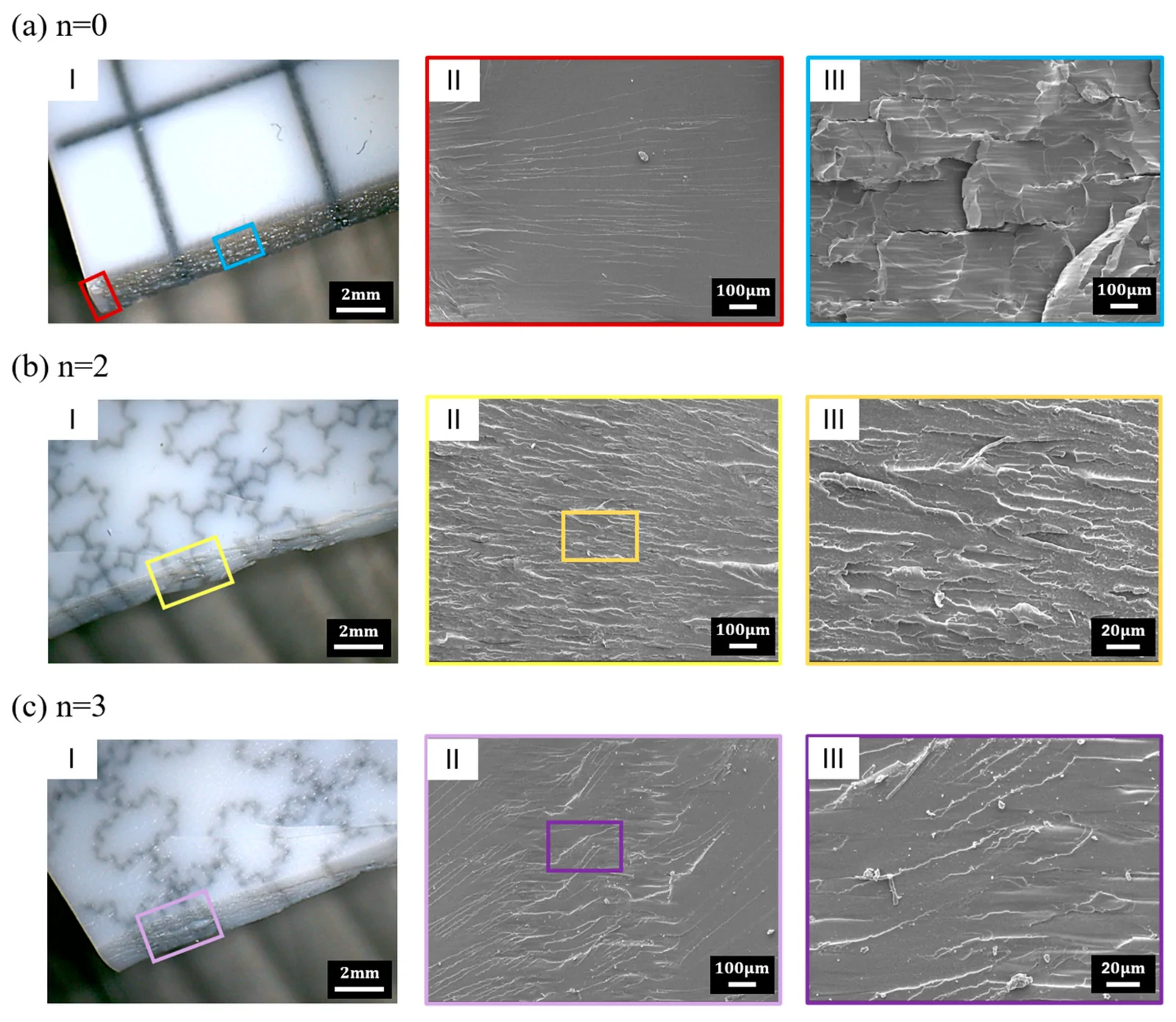
Fracture-surface morphology at selected fractal orders. (**a**) *n* = 0: (I) optical image locating the rigid- and compliant-phase observation regions, (II) comparatively smooth rigid-phase fracture surface, and (III) rougher compliant-phase fracture surface. (**b**) *n* = 2 and (**c**) *n* = 3: (I) optical images locating the SEM observation regions, (II) lower-magnification SEM images, and (III) enlarged views of the boxed regions in panel II. Overview fractographs in panels II and in ((**a**), III) were recorded at 100× magnification, and detailed views in ((**b**), III) and ((**c**), III) at 300× magnification. Boxes in panel I mark the SEM observation positions, boxes in panel II mark the regions enlarged in panel III, and the border color of each magnified panel matches its corresponding box. Scale bars: 2 mm in I; 100 μm in II and in ((**a**), III); 20 μm in ((**b**), III) and ((**c**), III).

Fractography supplied complementary evidence across length scales (Figure 6). For *n* = 0, the rigid-phase region was comparatively smooth, consistent with limited local deformation, whereas the compliant-phase region was rougher and showed twisting (Figure 6a). The *n* = 2 fracture surface contained elongated ligaments, step-like features, and tortuous traces, forming a pronounced high-relief morphology at the hard–soft interface that is consistent with repeated local crack deflection (Figure 6b). In contrast, the *n* = 3 surface exhibited finer, more parallel ridges and lower interfacial relief, consistent with a less tortuous fracture path (Figure 6c). The morphology therefore mirrors the macroscopic optimum: the *n* = 2 architecture combined pronounced interfacial relief with repeated deflection features, whereas geometric complexity alone at *n* = 3 did not guarantee more effective dissipation.

The elongated intact ligaments in the wake of the *n* = 2 crack (Figure 6b) are consistent with a secondary uncracked-ligament bridging contribution. This mechanism is distinguished from fiber pull-out or fiber bridging because the present composite contains no reinforcing fibers. The primary mechanism remains crack deflection and branching at modulus-mismatch interfaces, assisted by local crack-tip blunting in the compliant phase and by the distribution of strain over a broader region [3,61,62,63].

### 3.5. Pattern Orientation Governs the Strength–Deformation Balance

**Figure 7:**
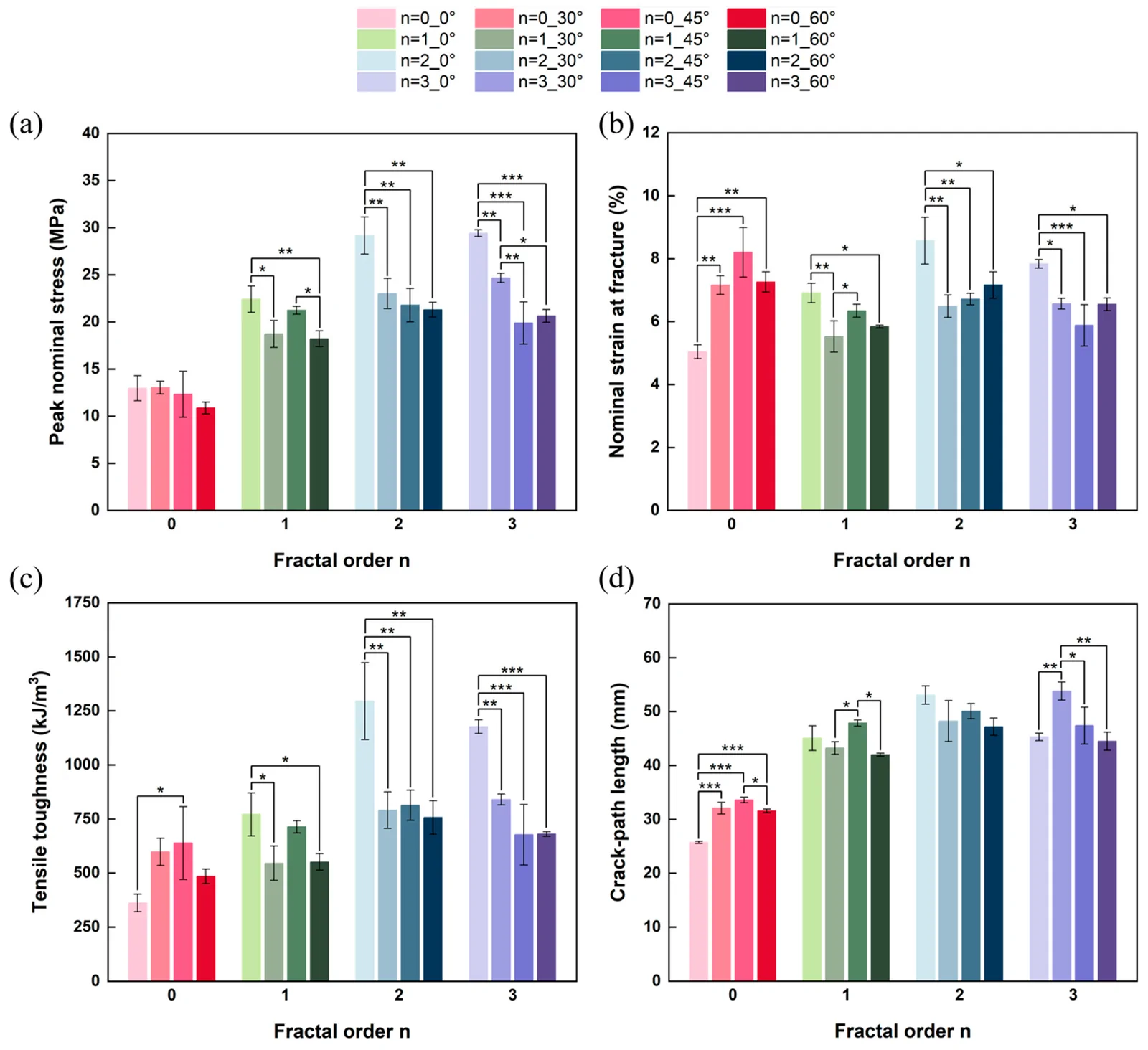
Combined effects of fractal order and pattern orientation on the pre-notched tensile response. (**a**) Peak nominal stress, (**b**) nominal strain at fracture, (**c**) tensile toughness, and (**d**) crack-path length. Bars show mean ± one standard deviation from three specimens per condition. Within each fractal order, brackets denote statistically significant pairwise differences among pattern orientations (* *p* < 0.05, ** *p* < 0.01, and *** *p* < 0.001).

Rotating the network changed the relationship between the nominal crack direction and the interfacial ligaments (Figure 7). For *n* = 0, the crack largely followed the compliant grid; rotation therefore redirected the path without materially increasing load-bearing capacity. In hierarchical specimens, orientation affected both the route and timing of branching. The 0° and 45° arrangements generally promoted earlier and wider branching, whereas 30° and 60° tended to guide the crack along a longer inclined route before separation. The *n* = 2 architecture produced the largest branching response at each orientation, indicating that orientation selects the direction of interaction while hierarchy determines how strongly that interaction is amplified.

Peak nominal stress showed the clearest orientation penalty at high order (Figure 7a). For *n* = 0, mean values were 13.0, 13.2, 12.3, and 10.9 MPa at *θ* = 0°, 30°, 45°, and 60°, respectively. The corresponding values were 22.4, 18.7, 21.2, and 18.2 MPa for *n* = 1; 29.2, 23.0, 21.8, and 21.3 MPa for *n* = 2; and 29.4, 24.7, 19.9, and 20.6 MPa for *n* = 3. Hierarchy improved peak nominal stress at every orientation, but its largest benefit occurred at 0°, where the ligament arrangement most effectively supported load transfer. Rotation could extend or incline the crack path, but that geometric benefit was frequently accompanied by lower peak nominal stress.

The remaining responses followed the same trade-off rather than a single universal optimum (Figure 7b–d). Rotated *n* = 0 specimens achieved larger nominal strains at fracture, greater tensile toughness, and longer inclined crack paths, although their stress level remained low. For *n* = 2 and *n* = 3, *θ* = 0° generally provided the largest area under the stress–strain curve, while selected rotated conditions increased path length or conditional *K*_C_. The *n* = 2 design maintained comparatively balanced performance across orientations, whereas *n* = 3 became competitive only for particular alignments. Pattern orientation should therefore be selected according to the target response: 0° for combined peak nominal stress and tensile toughness, or an inclined orientation when crack guiding or peak-load notch resistance is prioritized.

### 3.6. Conditional K_C_ and the Distinction Between Peak-Load Notch Resistance and Propagation

**Figure 8:**
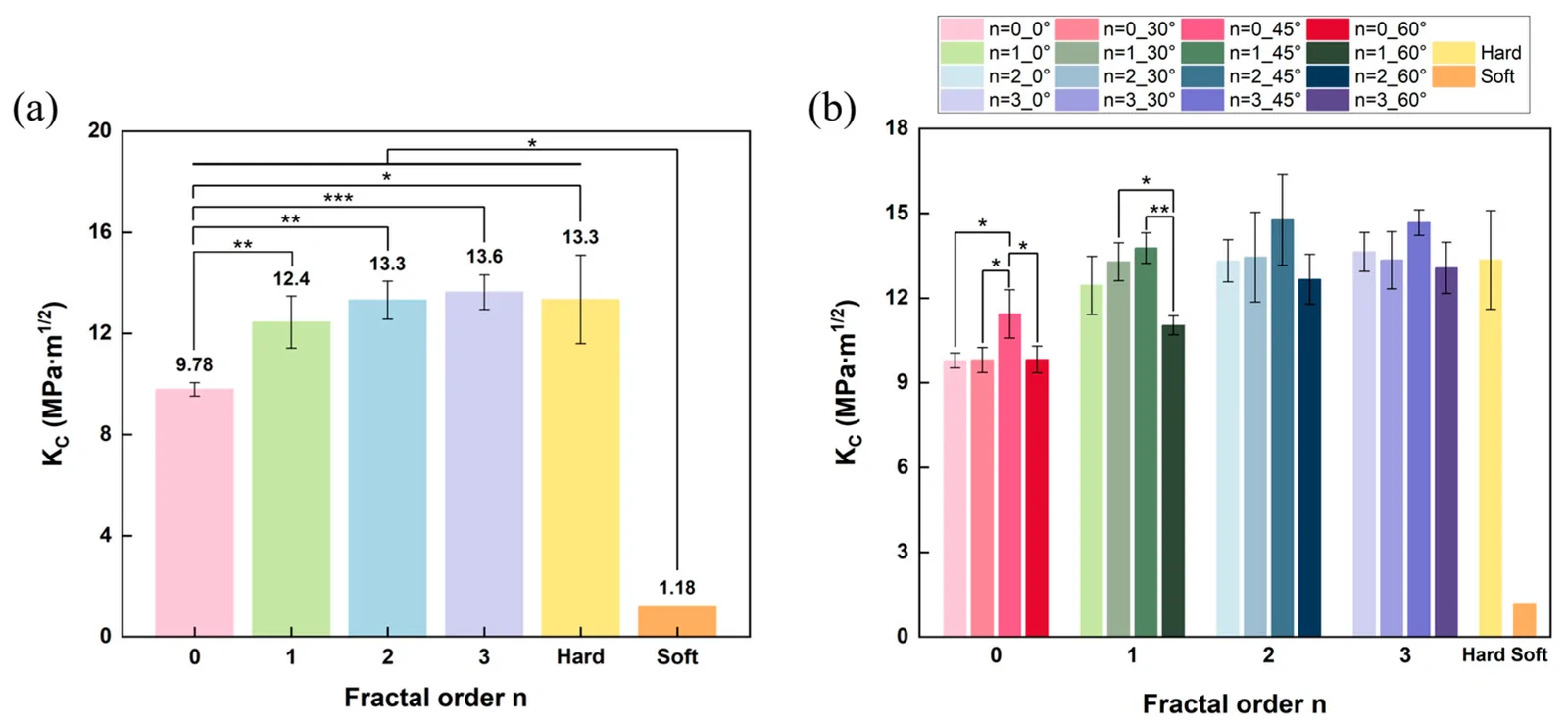
Conditional stress-intensity factor *K*_C_. (**a**) Fractal-order series at *θ* = 0° compared with rigid (Hard) and compliant (Soft) single-material references. (**b**) Combined effects of fractal order and pattern orientation, together with the single-material references. In (**a**), the *θ* = 0° composite groups and the two single-material references were analyzed as one comparison set. Bars show mean ± one standard deviation from three specimens per condition. Displayed brackets denote statistically significant pairwise differences (* *p* < 0.05, ** *p* < 0.01, and *** *p* < 0.001). *K*_C_ is reported as a conditional comparative parameter and is not a validated plane-strain *K*_IC_.

The conditional *K*_C_ of the single-material compliant reference was 1.18 MPa·m^1/2^, whereas that of the rigid reference was 13.3 MPa·m^1/2^ (Figure 8a). At *θ* = 0°, the composite value increased from 9.78 MPa·m^1/2^ at *n* = 0 to 12.4, 13.3, and 13.6 MPa·m^1/2^ at *n* = 1–3. Thus, *n* = 2 and *n* = 3 recovered a notch-resistance level comparable to the fully rigid material while retaining greater deformability. This comparison supports the architectural objective: the compliant phase need not cause a proportional loss of resistance when its interfaces are arranged to redirect and blunt the crack. Each composite group (*n* = 0–3) and the rigid single-material reference differed significantly from the compliant reference (*p* < 0.05).

The hierarchical groups *n* = 1–3 were statistically similar in *K*_C_ at *θ* = 0°, despite substantial differences in tensile toughness and crack-path length. This distinction is physically meaningful. Peak-load *K*_C_ is dominated by the local field near the notch and is treated here as a peak-load-based, geometry-dependent notch-resistance parameter. In contrast, tensile toughness integrates the subsequent loading and crack-growth history. The largest benefit of added hierarchy was expressed while the crack interacted with successive interfaces during propagation, not solely at the onset of fracture. A rising-resistance measurement was not performed, so the R-curve-type interpretation remains mechanistic rather than a measured resistance curve.

### 3.7. Orientation Dependence of Conditional K_C_

Conditional *K*_C_ varied jointly with fractal order and pattern orientation (Figure 8b). For *n* = 0, *K*_C_ was approximately 9.78–9.82 MPa·m^1/2^ at 0°, 30°, and 60° and increased to 11.4 MPa·m^1/2^ at 45°. For *n* = 1, values were 12.4, 13.3, 13.8, and 11.0 MPa·m^1/2^ at 0°, 30°, 45°, and 60°. The *n* = 2 series remained high across all orientations—13.3, 13.4, 14.8, and 12.7 MPa·m^1/2^—while *n* = 3 yielded 13.6, 13.3, 14.7, and 13.1 MPa·m^1/2^. The highest values occurred at 45° for *n* = 2 and *n* = 3.

The 45° maximum in Figure 8b indicates that an inclined near-tip ligament arrangement can increase the peak-load resistance of the notched geometry even when the same orientation does not maximize the integral tensile response. Conversely, the 0° design provided the strongest combination of peak nominal stress and tensile toughness for the hierarchical structures. Conditional *K*_C_ and tensile toughness should therefore be interpreted as complementary peak-load notch-resistance and global energy-density measures, respectively, together with the crack-path evidence for propagation behavior. This distinction explains why the 45° orientation maximizes the conditional *K*_C_ without maximizing tensile toughness: the former is governed by the near-notch stress field at peak load, whereas the latter integrates the deformation and propagation history of the entire patterned region. A comparable decoupling between peak-load fracture toughness and energy-based fracture measures has been reported for architected 3D-printed polymers, in which the printed geometry shifts the two quantities in different directions [64].

### 3.8. Toughening Mechanisms and Design Implications

**Figure 9:**
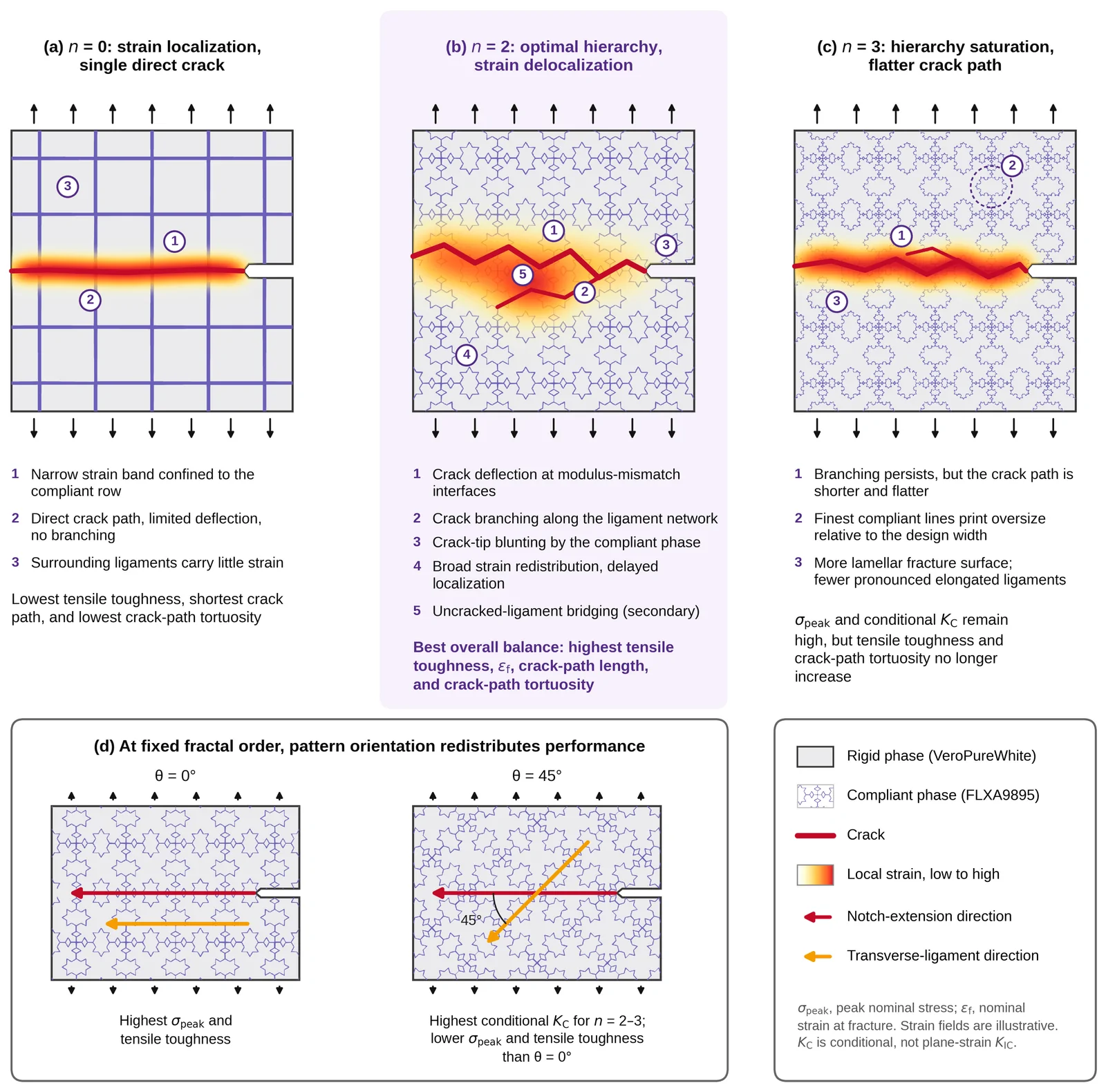
Schematic summary of hierarchy- and orientation-dependent toughening mechanisms. (**a**) The *n* = 0 network promotes a narrow strain-localization band and relatively direct cracking along the compliant row, resulting in limited deflection and low tensile toughness. (**b**) At *n* = 2, repeated interfacial crack deflection and branching, compliant-phase crack-tip blunting, broad strain redistribution, and secondary uncracked-ligament bridging provide the best overall balance of tensile toughness, nominal strain at fracture, crack-path length, and crack-path tortuosity. (**c**) At *n* = 3, nominal interface density increases, but local feature broadening, a more lamellar fracture surface, and less effective strain redistribution lead to diminishing returns; peak nominal stress and conditional *K*_C_ remain high, whereas tensile toughness and crack-path tortuosity no longer increase. (**d**) Pattern orientation redistributes performance at fixed fractal order: *θ* = 0° favors peak nominal stress and tensile toughness, whereas *θ* = 45° maximizes conditional *K*_C_ for *n* = 2–3 at the expense of peak nominal stress and tensile toughness. Rigid and compliant networks are rendered from the as-designed CAD geometry; strain fields are schematic and are not DIC data. *K*_C_ is a conditional comparative parameter and is not plane-strain *K*_IC_.

The results support a common mechanism in which compliant–rigid modulus mismatch is multiplied and spatially organized by the Koch-curve-derived network. When a crack reaches an interface, the local mismatch perturbs its stress field. The crack is redirected along or across the compliant region, its tip is blunted by local deformation, and the rigid skeleton continues to carry load. Repetition at multiple scales increases the probability of deflection and branching, lengthens the effective fracture route, and spreads deformation away from one narrow localization band. Elongated unbroken ligaments behind the *n* = 2 crack provide an additional shielding contribution before final rupture. These hierarchy-dependent mechanisms are summarized in Figure 9a,b.

Fractal order and orientation play distinct but coupled roles. Order controls the number and scale distribution of available interfaces; orientation controls how those interfaces are presented to the notch and applied load. The optimum is therefore not the architecture with the largest nominal complexity. At *n* = 2, interface density, rigid-skeleton continuity, and printable feature fidelity were sufficiently balanced to maximize crack tortuosity, strain redistribution, nominal strain at fracture, and tensile toughness. Among these mechanisms, repeated interfacial crack deflection and branching provides the prevailing contribution, as evidenced by the 2.1-fold crack-path elongation, with compliant-phase crack-tip blunting, strain redistribution, and secondary uncracked-ligament bridging acting as supporting contributions. At *n* = 3, peak nominal stress and conditional *K*_C_ remained high, but the fracture surface was less tortuous, strain localized earlier than at *n* = 2, and the smallest printed lines deviated from their design width, so that locally merged or poorly defined lines no longer acted as discrete crack-deflecting interfaces. The added hierarchy reached a regime of diminishing return under the present manufacturing conditions. In other words, *n* = 2 outperformed *n* = 3 not because recursive hierarchy ceased to be beneficial, but because the finest *n* = 3 features could no longer be printed with sufficient fidelity to function as designed. The contrast between the optimal and saturated hierarchical responses is illustrated in Figure 9b,c.

These findings lead to three practical design principles. First, maximize effective—not merely geometric—interface density while maintaining continuous load-bearing rigid ligaments. Second, align the architecture according to the desired failure response: 0° favored peak nominal stress and tensile toughness, while 45° maximized conditional *K*_C_ for the higher orders. Third, evaluate printing fidelity at the smallest recursive scale, because resolution-driven broadening changes local geometry and phase content. Among the tested designs, *n* = 2 at 0° was the best-performing configuration for balanced tensile fracture resistance, whereas *n* = 2–3 at 45° is preferable when peak-load notch resistance is the primary target. The orientation-dependent redistribution of performance is summarized in Figure 9d.

## 4. Conclusions

Koch-curve-derived compliant networks were co-printed within a rigid photopolymer matrix to determine how fractal order and pattern orientation govern tensile fracture. Introducing hierarchy increased peak nominal stress, nominal strain at fracture, tensile toughness, crack-path length, and conditional *K*_C_ relative to the *n* = 0 grid. At *θ* = 0°, *n* = 2 increased peak nominal stress, nominal strain at fracture, tensile toughness, and crack-path length by approximately 2.2-, 1.7-, 3.6-, and 2.1-fold, respectively.

The improvement did not increase monotonically with order. The *n* = 2 design produced the longest and most complex macroscopic crack path and the most pronounced ligament and step features on the fracture surface. Among the representative DIC fields for *n* = 0, 2, and 3, the *n* = 2 specimen showed the broadest strain zone and the latest defined strain-localization onset. The *n* = 3 design retained high peak nominal stress and conditional *K*_C_ but showed reduced printing fidelity and less effective overall deformation redistribution. Under the present material and manufacturing conditions, *n* = 2 therefore provided the best overall balance of strength and fracture resistance.

Pattern orientation changed how the ligament network interacted with the notch and load. The 0° orientation generally maximized peak nominal stress and tensile toughness for hierarchical structures, whereas 45° produced the highest conditional *K*_C_ for *n* = 2 and *n* = 3. Toughening was governed primarily by crack deflection and branching at modulus-mismatch interfaces, assisted by compliant-phase crack-tip blunting, distributed strain localization, and secondary uncracked-ligament bridging. The conditional *K*_C_ values are comparative and should not be interpreted as plane-strain *K*_IC_. The demonstrated architecture is relevant wherever damage tolerance must be combined with load-bearing capacity in printed polymer parts, for example protective housings and panels, energy-absorbing interlayers and mounts, compliant joints for soft robotics, and patient-specific biomedical devices that integrate stiff and soft regions in one part. The design principle is not restricted to material jetting: multimaterial vat photopolymerization, direct ink writing, and dual-nozzle fused filament fabrication could reproduce the architecture, subject to their respective feature-size, registration, and interfacial-bonding constraints. Future work should use fatigue-sharpened cracks or nonlinear fracture parameters, measure R-curves during stable growth, quantify build-direction effects, establish manufacturing-aware scaling rules for higher fractal orders, and characterize the response of the fractal hard–soft interfaces to cyclic and fatigue loading.

## Figures and Tables

**Table 1:** Materials, specimen geometry, and principal test conditions.

Category	Parameter	Condition
Constituents	Rigid phase	VeroPureWhite photopolymer
	Compliant phase	FLXA9895 digital material (nominal 90–96 Shore A)
Architecture	Fractal order	*n* = 0, 1, 2, and 3
	Pattern orientation, *θ*	0°, 30°, 45°, and 60°
	Soft-phase content	10% of the 30 × 30 mm patterned region (design value)
Specimen	Overall dimensions	80 × 31 × 2.3 mm
	Tensile notch	5.5 mm depth × 1 mm opening width
Tensile test	Nominal strain rate	1 × 10^−4^ s^−1^
Conditional *K*_C_ test	ESE(T)-type geometry	B = 2.3 mm; W = 31 mm; a = 15.5 mm; notch opening width = 5 mm; *a*/*W* = 0.5
	Nominal strain rate	6.67 × 10^−4^ s^−1^
Replication	Mechanical tests	Three specimens per condition
	DIC	One representative specimen per condition

**Table 2:** Designed and measured ligament widths, relative deviations, and estimated soft-phase fractions of the Koch-curve-derived networks.

*n*	*w_d_* (μm)	*w_m_* (μm)	*Δw* (%)	*φ*_*s*,*est*_ (%)
0	310	308.83 ± 6.45	−0.4	10.0
1	155	155.74 ± 4.83	+0.5	10.0
2	107	110.26 ± 3.63	+3.0	10.3
3	73.5	81.41 ± 6.49	+10.8	11.1

Symbols: *w_d_*, designed ligament width; *w_m_*, measured ligament width; *Δw* = (*w_m_* − *w_d_*)/*w_d_* × 100%, relative width deviation; *φ_s_*_,*est*_, estimated soft-phase fraction. Measured widths are mean ± one standard deviation from 30 measurements per fractal order (three specimens and ten measurements per specimen). The estimated soft-phase fraction is a first-order width-based estimate at fixed centerline length.

## Data Availability

The data presented in this study are available from the corresponding author upon reasonable request.
